# Comunicação da PrEP e da PEP no Brasil: exploração dos sentidos de peças comunicacionais e análise das concepções de agentes governamentais

**DOI:** 10.1590/0102-311XPT100524

**Published:** 2025-10-03

**Authors:** Claudia Mora Cárdenas, Simone Souza Monteiro

**Affiliations:** 1 Instituto de Medicina Social, Universidade do Estado do Rio de Janeiro, Rio de Janeiro, Brasil.; 2 Instituto Oswaldo Cruz, Fundação Oswaldo Cruz, Rio de Janeiro, Brasil.

**Keywords:** Profilaxia Pré-exposição, Profilaxia Pós-exposição, HIV, Comunicação, Simbolismo, Pre-exposure Prophylaxis, Post-exposure Prophylaxis, HIV, Communication, Symbolism, Profilaxis Pre-exposición, Profilaxis Posexposición, VIH, Comunicación, Simbolismo

## Abstract

Informado pelas críticas sobre o fim do paradigma da excepcionalidade nas respostas à aids no Brasil, o trabalho analisa as concepções e práticas de comunicação sobre prevenção do HIV por parte de agentes governamentais e suas implicações simbólicas e programáticas. A reflexão integra uma pesquisa mais ampla com usuários, profissionais e gestores de cinco programas municipais de HIV e/ou aids da Região Metropolitana do Rio de Janeiro, Brasil, e do Governo Federal. A partir das contribuições das ciências sociais para o entendimento das representações e práticas sociais em saúde, a investigação envolveu: análise de entrevistas com gestores (federais e locais) e profissionais de saúde acerca das estratégias de comunicação da prevenção combinada (PC) e das profilaxias pré-exposição (PrEP) e pós-exposição (PEP) ao HIV; e análise de 24 peças de comunicação sobre PC, PrEP e PEP. Os agentes governamentais relataram estratégias de divulgação da PrEP e PEP para os profissionais de saúde, por meio de material de consulta e oficinas, frente à rotatividade desse grupo e às resistências de cunho moral e ético-político. Porém, a divulgação pública das profilaxias é discreta ou se dá no plano digital. A exploração dos sentidos das peças de comunicação indica uma ênfase na dimensão clínica das profilaxias nos materiais governamentais e nas peças das organizações não-governamentais, em que há maior contextualização das estratégias de PC para as cenas, práticas e identidades sexuais. Nota-se um deslizamento das estratégias de comunicação para uma gramática informada pela disponibilidade de biotecnologias e sua enunciação dispersa. A fragilização da comunicação governamental na era da PC compromete a efetivação do direito à prevenção.

## Abstract (en)

This study analyzes the conceptions and communication practices of government agents on HIV prevention and their symbolic and programmatic implications based on the criticism regarding the end of the paradigm of exceptionality in AIDS responses in Brazil. This reflection is part of broader research with users, professionals and managers of five municipal HIV/AIDS programs carried out in the Metropolitan Area of Rio de Janeiro, Brazil, and run by the Federal Government. Based on contributions of the social sciences to the understanding of social representations and practices in health, the investigation involved an analysis of interviews with managers (federal and local) and health professionals about the communication strategies of combined prevention (CP), HIV pre-exposure (PrEP) and post-exposure (PEP) prophylaxis and an analysis of 24 pieces of communication on CP, PrEP and PEP. Government agents reported strategies for disseminating PrEP and PEP to health professionals via consultation material and workshops, considering the turnover of this group and moral and ethical-political resistance. However, the public disclosure of prophylaxis is discreet or carried out digitally. The exploration of the meanings of the communication pieces indicates an emphasis on the clinical dimension of prophylaxis in government materials; in pieces created by nongovernmental organizations a greater contextualization of CP strategies for sexual scenes, practices, and identities was found. A shift from communication strategies to a grammar informed by the availability of biotechnologies and their dispersed enunciation was observed. The weakening of government communication in the era of CP compromises the effectiveness of the right to prevention.

## Abstract (es)

Basándose en las críticas sobre el fin del paradigma de la excepcionalidad en las respuestas al HIV en Brasil, este trabajo analiza las concepciones y prácticas de comunicación sobre la prevención del VIH de los agentes gubernamentales y sus implicaciones simbólicas y programáticas. La reflexión forma parte de una investigación más amplia con usuarios, profesionales y gestores de cinco programas municipales de VIH/SIDA de la Región Metropolitana de Río de Janeiro, Brasil, y del Gobierno Federal. Desde de la mirada de las ciencias sociales para la comprensión de las representaciones y prácticas sociales en salud, la investigación incluyó: el análisis de entrevistas con gestores (federales y locales) y profesionales de salud sobre las estrategias de comunicación de la prevención combinada (PC) y los profilaxis de pre-exposición (PrEP) y post-exposición (PEP) al VIH; el análisis de 24 piezas de comunicación sobre PC, PrEP y PEP. Los agentes gubernamentales difundieron las estrategias de PrEP y la PEP entre los profesionales de salud, mediante materiales de consulta y realización talleres, ante la alta rotación de este grupo y las resistencias de carácter moral y ético-político. Sin embargo, la divulgación pública de estas profilaxis es discreta o se limita al ámbito digital. El análisis del significado de los materiales de comunicación indica que en los materiales gubernamentales predomina un enfoque clínico de las profilaxis, mientras que los materiales de las organizaciones no gubernamentales contextualizan en mayor medida las estrategias de PC para las situaciones sexuales, las prácticas y las identidades sexuales. Se observa un desplazamiento de las estrategias comunicacionales hacia un lenguaje centrado en la disponibilidad de biotecnologías, con una enunciación dispersa. El debilitamiento de la comunicación gubernamental en la era de la PC compromete la efectividad del derecho a la prevención.

Estratégias de comunicação massiva, marketing e campanhas, visando à promoção de práticas e ao uso de biotecnologias de cunho preventivo, fazem parte do marco de ações e discursos inscritos na denominada Nova Saúde Pública ^1^. Sob uma perspectiva teórica, que articula saúde e comunicação, interessa compreender as representações relativas a público, contexto, comportamentos e insumos que engendram esses investimentos ^2^ ^,^ ^3^ ^,^ ^4^ ^,^ ^5^. Essa abordagem tem sido fundamental para interpretar os sentidos e significados que as “*tecnologias educacionais produzem*”, como fôlderes, cartilhas, cartazes ^6^ (p. 20), incluindo aquelas que circulam nas mídias sociais.

Desde o surgimento dos primeiros casos de aids, no início da década de 1980, foram produzidas pesquisas focadas na circulação e interpretação de cartazes, matérias jornalísticas e campanhas preventivas, dentro e fora do país ^6^ ^,^ ^7^ ^,^ ^8^. São também ilustrativas as contribuições de estudos nacionais, no plano das representações, sobre a infecção e os sujeitos envolvidos nas cenas retratadas ^9^, assim como no âmbito das políticas públicas em torno das estratégias de informação e comunicação ^10^. Todavia, são escassas as pesquisas relativas à concepção, produção e difusão das estratégias de comunicação, que fazem parte do enfrentamento atual da epidemia no contexto brasileiro ^11^. 

Importa ressaltar que, na década de 2010, houve mudanças nas diretrizes globais que envolvem a disponibilização de novas biotecnologias de prevenção, enunciadas por especialistas e agências internacionais que vislumbram o fim da epidemia em 2030 ^12^ ^,^ ^13^. Assim, a estratégia da prevenção combinada (PC) se consolida sob a premissa de articular intervenções biomédicas, estruturais e comportamentais segundo os contextos locais e regionais ^14^. No escopo da PC, tem havido investimentos, especialmente no diagnóstico precoce do HIV e no encaminhamento dos casos de HIV positivo, para o tratamento, por meio da ampliação do acesso ao teste rápido e à terapia antirretroviral (TARV), conhecido como tratamento como prevenção (TcP). 

Outra frente refere-se à oferta da profilaxia pré-exposição (PrEP) ao HIV, caracterizada pelo uso regular de antirretrovirais antes da exposição ao HIV, e da profilaxia pós-exposição (PEP), para casos de acidente profissional, violência sexual e sexo desprotegido, que deve ser usada até 72 horas após a exposição ao HIV nos serviços de emergência ^15^. Ambas são disponibilizadas pelo Sistema Único de Saúde (SUS). Inicialmente, a PrEP era indicada para casais sorodiscordantes e populações com maior prevalência de HIV (homens que fazem sexo com homens - HSH, pessoas transgênero e trabalhador(a) do sexo). Entretanto, na versão revisada do protocolo clínico e diretrizes terapêuticas de 2022, a PrEP passou a ser passível de indicação para qualquer pessoa em situação de vulnerabilidade para o HIV ^16^. Uma avaliação de contextos e práticas parece ganhar espaço como critério de inclusão, embora a estratégia continue sendo recomendada para as populações-chave que apresentem risco aumentado de infecção. No caso da PEP, o foco permanece nos contextos de exposição ao vírus, independentemente de aspectos identitários dos sujeitos envolvidos.

As estratégias supracitadas (TcP, PrEP e PEP) são alavancadas por investimentos contínuos por parte de uma teia de agentes, integrada por redes de pesquisa biomédica, organizações da sociedade civil, governos, agências de saúde internacionais e organizações filantrópicas ^17^ ^,^ ^18^. 

Diante desse panorama global e sua sedimentação no cenário nacional e local, este trabalho buscou compreender as concepções acerca das estratégias de comunicação da PrEP e da PEP. Para tanto, priorizou-se a perspectiva de agentes governamentais, no âmbito federal e municipal, e as mensagens das peças de comunicação sobre as novas profilaxias, que circulam nos espaços governamentais de assistência e prevenção ao HIV. O estudo integra uma pesquisa mais ampla (*Biomedicalização da Resposta à Aids: O Acesso de Gays, Mulheres Trans/Travestis e Prostitutas às Profilaxias Pré e Pós Exposição na Região Metropolitana do RJ*), desenvolvida entre 2019 e 2021 sobre a implementação da PrEP e da PEP nos serviços de saúde e as experiências de uso em municípios da Região Metropolitana do Estado do Rio de Janeiro, Brasil. O trabalho de campo envolveu pesquisa documental, observação nos serviços, entrevistas com usuários (gays, mulheres trans e/ou travestis, trabalhadoras sexuais), gestores, profissionais de saúde e lideranças comunitárias.

A presente investigação partiu da hipótese de que a divulgação e circulação das informações sobre prevenção e cuidado ocorrem de maneira pulverizada. Diversos atores participam da sua oferta, com perspectivas e linguagens não necessariamente uníssonas. Desse modo, é importante identificar sob uma perspectiva sociológica, as visões de atores governamentais envolvidos nas ações de comunicação das profilaxias e as especificidades do contexto sócio-histórico que influenciam sua disseminação ^19^.

## Metodologia

Trata-se de um estudo qualitativo, informado pelas contribuições das ciências sociais no entendimento das relações, simbolismos e valores presentes nas representações e práticas sociais no campo da saúde ^20^. Após aprovação pelo Comitê de Ética em Pesquisa da Fundação Oswaldo Cruz (FIOCRUZ; CAAE: 16076619.2.0000.5248), o estudo envolveu três procedimentos complementares. O primeiro refere-se à realização de entrevistas via telefone, com duas gestoras que atuaram no período de implementação da PrEP (2017-2018); uma na direção do então Departamento de Aids/IST e Hepatites Virais do Ministério da Saúde; e a outra, que desde então coordena uma das áreas desse departamento, atualmente denominado Departamento de HIV, Aids, Tuberculose, Hepatites Virais e ISTs. 

A segunda etapa diz respeito às ações de comunicação relatadas por profissionais responsáveis pela implementação das novas estratégias preventivas do HIV em cinco municípios da Região Metropolitana do Rio de Janeiro, sendo eles: Niterói, Rio de Janeiro, Duque de Caxias, São Gonçalo e Nova Iguaçu. No momento da pesquisa, a PEP havia sido implementada em serviços de emergência, e a PrEP, até então, em apenas cinco unidades no estado. Após o contato da equipe do projeto com as coordenações municipais de IST/HIV/aids das cinco localidades e da assinatura do Termo de Consentimento Livre e Esclarecido foram entrevistados seis profissionais de saúde dos serviços de PrEP e PEP e quatro gestores(as) municipais. Como indica o Quadro 1, os(as) entrevistados(as) são majoritariamente médicos(as) infectologistas e enfermeiras(os), enquanto outros têm formação em serviço social. Todas as entrevistas foram gravadas e realizadas presencialmente nos locais de trabalho dos interlocutores.

**Quadro 1:** Formação e cargo dos gestores e profissionais entrevistados.

CÓDIGO	CARGO	MUNICÍPIO	FORMAÇÃO PROFISSIONAL
1GMFe	Gestão municipal	Rio de Janeiro	Medicina
2GMFe	Gestão municipal	Rio de Janeiro	Enfermagem
3GMFe	Gestão municipal	Niterói	Enfermagem
4GMFe	Gestão municipal	Duque de Caxias	Medicina
5PRFe	Profissional de serviço especializado	Duque de Caxias	Serviço Social
6AAFe	Assistente administrativo de serviço PrEP	Rio de Janeiro	Não informado
7CSFe	Coordenação de serviço PrEP	Niterói	Enfermagem
8CAMa	Coordenação ambulatório hospitalar HIV/aids e PEP	Nova Iguaçu	Medicina
9PRFe	Profissional de serviço PrEP e PEP	São Gonçalo	Enfermagem
10CSFe	Coordenação de serviço PrEP	Rio de Janeiro	Enfermagem

Fe: mulher; Ma: homem; PEP: profilaxia pós-exposição; PrEP: profilaxia pré-exposição.

A análise das entrevistas se fundamentou no método de interpretação dos significados sociais ^21^, a partir da leitura das informações, organização e codificação do conteúdo. O processo resultou nas seguintes categorias temáticas: simbolismos das campanhas, concepções sobre prevenção, expectativas das estratégias de divulgação em termos de regulação das sexualidades, garantia do direito à prevenção da aids e visões e experiências dos interlocutores. Foram priorizadas as convergências e tensões entre os agentes envolvidos na produção de estratégias de comunicação, visando produzir um panorama dos aspectos políticos, sociais e institucionais dos simbolismos e efeitos práticos das respostas atuais à epidemia. 

A terceira etapa envolveu a análise exploratória de 24 peças de comunicação impressas sobre PrEP, PEP e PC, produzidas por instituições governamentais e pelo movimento social local. Todas foram coletadas durante as visitas dos(as) pesquisadores(as) aos serviços e organizações não-governamentais (ONGs) dos cinco municípios referidos. A análise desses materiais objetivou identificar as representações sobre os sujeitos e estratégias preventivas, as instituições produtoras e informações de contatos ou locais para encaminhamento, não sendo priorizada a variação do formato dos materiais. Para tanto, foi estruturada uma matriz descritiva contendo: tipo de material (cartaz, folheto, fôlder etc.); instituição produtora (estado, sociedade civil, instituição de pesquisa, coalizão interinstitucional); tema (PC, PrEP, PEP); público; mensagens escritas; e imagens, cores e símbolos. 

## Resultados e discussão

### A perspectiva de gestores (nacionais e locais) e profissionais

Ao longo da história da aids, foram produzidas diversas campanhas preventivas, divulgadas durante o ano e intensificadas no Dia Internacional de Luta contra à Aids (1º de dezembro), no carnaval e em outras festividades regionais. Tal enfoque contrasta com as estratégias de disseminação governamentais identificadas até 2022, nas quais a circulação de informações sobre as novas estratégias de prevenção é relativamente restrita, com foco em determinados sujeitos e redes de colaboração nos níveis local, regional e nacional. 

Essa afirmação se sustenta na revisão das diretrizes nacionais para a expansão da PrEP na rede de serviços de saúde e nos relatos colhidos. O documento *Orientações para a Expansão da Oferta da Profilaxia Pré-Exposição (PrEP) ao HIV na Rede de Serviços de Saúde* ^22^ (p. 9), de 2018, contempla a dimensão da comunicação em termos de “*mobilizar a comunidade local (redes e ONG)*”. Todavia, as denominadas estratégias de informação, educação e comunicação ou a previsão de uma ampla divulgação não foram referidas. 

Os depoimentos das gestoras do nível federal, por sua vez, indicam que as estratégias de divulgação das novas profilaxias respondem a uma lógica semelhante ao “escalonamento”, caracterizada pela distribuição de cartazes e fôlders da federação para os estados e municípios. Essa prática não é novidadeira e, de fato, constitui uma alternativa especialmente relevante em contextos locais onde a alocação ou execução de recursos para a confecção de materiais próprios não é algo priorizado. As interlocutoras não detalharam os processos de produção desses materiais, nem aprofundaram o impacto da sua divulgação através das redes sociais governamentais. Chama atenção ainda que naquele momento de implementação das profilaxias (2017-2018), na lógica da gestão federal, as peças comunicacionais eram dirigidas exclusivamente para o público destinatário delas. Ou seja, os materiais da PrEP para as populações chave e casais sorodiscordantes e os da PEP para a população em geral. 

Em suma, os depoimentos sugerem que a produção e circulação de campanhas e materiais informativos, voltados para a implementação e acesso à PrEP e à PEP, não têm sido informadas pela valorização dos elementos subjetivos, políticos, culturais e institucionais da comunicação ^3^ ^,^ ^5^. As evidências podem ser compreendidas por um certo apagamento da aids como um problema social, na última década, agudizado pela pandemia de COVID-19, pelo recrudescimento do discurso conservador e pelo desmonte das políticas de saúde sexual e reprodutiva e direitos humanos do Governo Federal de 2019 a 2022. São ilustrativos os pronunciamentos estigmatizantes contra as pessoas que vivem com HIV e aids, por parte do então Presidente - Jair Messias Bolsonaro -, e os efeitos deletérios do desmonte da Secretaria de Comunicação Social da Presidência da República (SECOM), que funcionou de forma inconsistente e desarticulada, especialmente no plano da comunicação digital. 

No âmbito local, as entrevistas com profissionais e gestores(as) dos municípios de São Gonçalo, Duque de Caxias, Niterói, Rio de Janeiro e Nova Iguaçu, somadas à análise do contexto programático de prevenção e cuidado do HIV e/ou aids desses municípios e das condições de vulnerabilidade ao HIV vividas por gays, transsexuais, travestis e prostitutas, descritas em artigo anterior ^23^, revelam as nuances da divulgação e ilustram os diferentes fios que constituem o emaranhado da circulação de informações oficiais da PEP e da PrEP. Com base na noção de “*poder simbólico*” ^24^ (p. 14), no qual os agentes detentores do poder mobilizam seus recursos para “*fazer ver, fazer crer*” ^24^ (p. 14), exploramos os modos como os interlocutores relatam ter mobilizado recursos para tornar visível o modelo preventivo em voga. Em meio à precarização administrativa e à normalização da aids na esfera pública ^18^, os gestores contam com estratégias e atores diversificados, elencados a seguir. 

Do ponto de vista da gestão, as estratégias de comunicação para divulgação dos protocolos e demais diretrizes acerca das novas tecnologias começou pelas equipes de profissionais de saúde. Dada a alta rotatividade desse grupo na rede pública, essas ações são qualificadas como um “*trabalho de formiguinha*”. Para isso, os(as) interlocutores(as) relataram a produção de materiais de consulta, os quais recebem formas distintas de enunciação (“*combo de informações*”; “*guia rápido para profissionais*” ou “*bibliazinha*”; “*folheto PEP-PrEP*”; “*caderneta da diversidade sexual*”) e que circularam em paralelo à incorporação do tema nas oficinas, rodas de conversa e eventuais capacitações sobre ISTs. Por vezes, tais ações ocorriam de modo protocolar (“*via ofício mesmo*”) e descontinuada (“*de vez em quando, quando é ocasião assim, dia mundial de combate da sífilis, aí a gente fala do serviço*”).

Esse empreendimento parece priorizar os atores e dinâmicas dos serviços sob o mote: “*fazer com que as pessoas ouvissem falar do que era PEP* (...) *o que era PC*”. Para a gestão do Município do Rio de Janeiro, frente aos desafios do modelo de descentralização das ações de assistência e prevenção da aids, a produção de uma caderneta de bolso sintetiza “*tudo que pode ser feito e que o município está fazendo*”, concretizando os esforços da gerência de HIV. A produção de estratégias de comunicação e informação com foco nos profissionais visou à apropriação dos protocolos das profilaxias, como relatado:

“...*tem que ser um material que esteja atualizado… para que ele possa fazer uma intervenção rápida e não desassistir esse paciente por conta das 72 horas para garantir o tratamento*” (2GMFe). 

O alcance dessa estratégia é atravessado por resistências de cunho ético-político, nem sempre explícitas, que expressam as restrições de cunho moral na oferta de tecnologias preventivas para grupos sociais tidos como “desviantes” (gays, mulheres trans e profissionais do sexo). Para as interlocutoras de Niterói e de São Gonçalo, não foram poucos os empecilhos da implementação da PrEP entre profissionais tidos como “conservadores”. Eles alegavam aumento da carga de trabalho ou advertiam que a oferta da profilaxia desencadearia o temido “*abandono do preservativo*”. Em Duque de Caxias, os médicos pouco se envolveram nos treinamentos alegando falta de tempo. Paradoxalmente, enfermeiros(as) da região participaram do processo, apesar de terem maior rotatividade. Essa visão é corroborada pela gestão do município do Rio de Janeiro, na medida em que:

“...*ainda existe uma postura muito rígida dos profissionais, de julgamento, preconceito, intolerância com relação à diversidade, o que reduziria a possibilidade de acesso das populações que mais precisam*” (1GMFe).

Assim, identificamos alguns indícios das moralidades em jogo na esfera local na implementação das profilaxias, as quais se enlaçam à dinâmica do par “esperança-medo”, que perpassou a discussão pública da PrEP. Em especial na cena da sua aprovação como estratégia sanitária na esfera federal e na sua implementação, como analisado por Brigeiro & Monteiro ^18^. Esse processo, aparentemente, distingue-se da discussão em torno da PEP sexual em termos temporais, ocorrida antes de 2010, e não foram encontradas pistas nos documentos e entrevistas com as gestoras do nível federal. Ademais, havia um maior distanciamento do contexto de profusão de pânicos morais, somados à discriminação em relação a sexualidade, orientação sexual e identidade de gênero, sob o amparo de argumentos supostamente técnicos. 

Na conclusão da pesquisa de campo, em fevereiro de 2020, a PEP na cidade do Rio de Janeiro estava sendo assumida pelas unidades de atenção básica, devido ao processo de descentralização. Contudo, a maior parte do seu acesso na zona metropolitana ocorria nos serviços de emergência dos hospitais e nas unidades de pronto atendimento (UPAs), uma vez que a PEP precisa estar disponível 24 horas, todos os dias da semana. De qualquer modo, os sentidos da emergência para quem procura a PEP não convergem com os serviços ambulatoriais onde há dispensa, como relatado por um médico de Nova Iguaçu: 

“*A queixa que ouço é: ‘A gente ficou rodando aí. Ninguém sabia o que fazer ou todo mundo falou que era pra vir aqui. Era final de semana’. Ouvi reclamação pelo fato de não abrir aos sábados. Mas não é pra abrir. Nunca abri*” (8CAMa).

Quanto à divulgação da PEP e da PrEP destinada aos usuários(as), parte dos(as) gestores(as) e profissionais do Rio de Janeiro considera que as estratégias de comunicação são abrangentes em termos da organização da rede. Distinto do que preconizam as reflexões no campo da comunicação em saúde ^3^ ^,^ ^4^ ^,^ ^5^, houve poucas apreciações acerca dos investimentos na disseminação e apropriação das informações por parte dos potenciais usuários. Segundo um profissional, os fôlderes distribuídos no serviço eram “*simples e objetivos: para gregos e troianos*”, tendo sido produzidos por uma instituição de pesquisa. 

Os registros do trabalho de campo atestam a pouca visibilidade da oferta das profilaxias. Em uma unidade de alta demanda de PrEP, a porta principal possuía apenas um cartaz: *Prevenção*, seguido da indicação *Entre sem bater*. Nos demais locais, predominavam ambientes decorados com cartazes de campanhas anteriores. Ocasionalmente, foi observado um cartaz da indústria farmacêutica sobre os efeitos colaterais dos antirretrovirais. 

O histórico de produção de campanhas e distribuição de peças de comunicação por parte do Ministério da Saúde, por vezes em articulação com a sociedade civil, possivelmente representou a principal fonte de divulgação das estratégias da resposta estatal à aids. Entretanto, a aparente erosão dessa esfera por parte do executivo na era da PC contribui para a mobilização de outros atores no intuito de “*fazer ver*” as profilaxias. O relato de uma assistente social converge com essa impressão ao descrever o processo de divulgação da PEP:

“*Se não me engano, foi na mídia. Nós até botamos panfletos, cartazes divulgando alguma coisa, mas era uma coisa muito fraquinha. A mídia que divulgou bastante sobre ‘rompeu a camisinha? Procura uma emergência’, e aí chegamos na emergência e eles mandam pra gente, pro segundo atendimento*” (5PRFe).

Uma fonte de divulgação imprecisa, mas frequentemente evocada, foi a internet, as redes sociais institucionais dos equipamentos de saúde foram pouco lembradas. Por vezes, os profissionais referem-se a grupos que discutem o tema nas redes sociais, indicam sites específicos de ONGs de referência ou parcerias com equipamentos das políticas de cidadania LGBTQIA+, como o Centro de Cidadania LGBT do Município de Duque de Caxias. 

Ainda no escopo das alianças locais, para a divulgação das profilaxias e demais ações preventivas, as ONGs foram citadas como o trabalho de “*conscientização*” feito pelo Grupo Diversidade de Niterói. Todavia, foi ponderado que sua ação é circunscrita a determinadas redes de sociabilidade: “*divulgação dentro da bolha*” via educadores de par. Tal enfoque contrasta com o papel histórico dos movimentos sociais nas respostas à epidemia e no controle social ^18^. Equipamentos do Sistema Único de Assistência Social, como os Centros de Referência da Assistência Social (CRAS) também compõem o elenco de parcerias para o encaminhamento de pessoas transgêneros em São Gonçalo. Da mesma forma, a Coordenadoria de Diversidade Sexual no Rio é tida como instituição parceira, capaz de mobilizar recursos e articulações para a gestão do município.

Por fim, o repasse de informações “boca a boca” acerca da disponibilidade de vagas para PrEP nos locais de dispensação parece dinamizar o fluxo das informações entre usuários e potenciais usuários:

“*Hoje a gente não precisa de mais divulgação, se a gente abrir, vai ter paciente. Eu não sei como isso aconteceu, entendeu?*” (5PRFe).

Essa enunciação ilustra como nas respostas biomédicas os sujeitos da prevenção não apenas são enquadrados sob o rótulo “paciente”, mas se esvazia a ideia do sujeito da prevenção na sua dimensão coletiva ou comunitária. No momento da pesquisa, anterior à COVID-19, a gestão dos municípios não previa estratégias para garantir a divulgação de informações sobre a PC para o público (geral ou específico). Desse modo, a própria experiência com as profilaxias suscita em alguns sujeitos a vontade de repassar informações nos seus espaços de sociabilidade ^25^.

Um aspecto relevante sobre o alcance das estratégias de comunicação, relatado pelos interlocutores e atestado pela literatura, refere-se ao perfil dos usuários da PrEP nos cinco municípios. Eles são majoritariamente gay/HSH, brancos, com renda e escolaridade média ou superior. As mulheres transsexuais e/ou travestis participam em menor proporção e chegam via educador de par, articulações interinstitucionais junto aos equipamentos da assistência social, como os CRAS ou instituições de pesquisa. A procura espontânea é ainda menos frequente entre as mulheres profissionais do sexo. As desigualdades no acesso às profilaxias são de difícil enfrentamento quando os moldes das ações estão sujeitos principalmente ao critério de lotação dos serviços. Mesmo assim, vários gestores reconhecem com preocupação, como exemplificam as afirmações: “*quem está mais informado é quem menos precisa das tecnologias*”; “*no município não acho que a PrEP consegue atingir quem realmente precisa*”. 

A previsão de estratégias de comunicação por parte dos interlocutores emerge como uma ação menos urgente e desprovida de consenso acerca de seus princípios ou relevância. Nesse sentido, cumpre elencar as distintas concepções em jogo sobre o que é fazer prevenção na visão das gestoras locais do Rio de Janeiro e de Duque de Caxias, quem expressaram alguns dilemas, respectivamente: 

“*Antigamente, se realizava prevenção tendo como preocupação o comportamento, autoconsciência com relação ao corpo, às práticas e riscos.* (...) *e também os preservativos* (...)*. A aids foi ficando mais simples e mais comum* [com antirretrovirais mais eficazes, disponíveis e simplificados]*. Ainda acredito que temos que voltar a trabalhar o comportamento, principalmente com a população mais jovem, para que as escolhas sejam mais conscientes e para que a gente precise menos de lançar mão de questões mais extremas ou medicamentosas como PEP e PrEP*” (1GMFe).

“*Tinha que ter mais ações educativas, só a PrEP não é o ideal. O CTA podia ter movimento maior, não de pessoas se testando, mas pessoas procurando informação, o que não acontece. Com a PrEP, a gente estava começando mais um serviço, não atuando em prevenção*” (4GMFe).

Essas reflexões indicam o interesse em desenvolver outras ações, além das intervenções medicamentosas, previstas na diretriz nacional da PC, atestando uma tensão entre o tempo da prevenção e o tempo da prescrição (das profilaxias). Isto é, na configuração atual dos equipamentos da prevenção da aids, só haveria espaço para realizar parte do modelo preventivo oficial:

“*A PC é um ‘papo sobre as possibilidades’. Eu vou ter que conversar com alguém e entender primeiro quem é esse alguém, e oferecer para ele as possibilidades, entendendo que algumas não vão servir para ele*” (1GMFe). 

As condições para desenvolver esse tipo de diálogo são um desafio para as interlocutoras. Esse dilema pode ser observado desde a expansão da testagem e sua dissociação do aconselhamento, entre os anos 2000 e 2010, momento em que a visão da “excepcionalidade” da aids é deslocada para uma visão “normalizadora” ^26^ ^,^ ^27^. 

O denominado tempo da prevenção diz respeito a diversos processos e canais de comunicação, como campanhas massivas, aconselhamento individual e coletivo, produção de peças audiovisuais, rodas de conversa etc. Nesse sentido, para além da visão dos gestores e profissionais, nos debruçaremos em um conjunto de peças de comunicação impressas sobre PrEP e PEP para entender os agentes e mensagens que circulam ou protagonizam a divulgação e comunicação das profilaxias no contexto pesquisado. 

### Peças comunicacionais: produtores e mensagens

Os materiais impressos corroboram a impressão da escassez de investimentos e de recursos públicos para a produção de material nas localidades. As instituições produtoras das 24 peças, segundo os logos institucionais dos materiais, são: ONG ou movimento social (12); Governo Federal (3); coalizão entre instituições públicas de pesquisa e Governo Federal (6); Governo Municipal (2); e editora particular (1). 

Nas peças de comunicação, cujos enunciadores são instituições governamentais, prevalecem mensagens voltadas para a dimensão clínica das estratégias de prevenção, baseadas nos antirretrovirais e ferramentas digitais (*websites*, redes sociais). Assim, espera-se que os receptores tenham motivações suficientes para procurar informações sobre o tema. Chama atenção como essa estratégia se caracteriza pela economia na produção de mensagens impressas constituídas por premissas, imperativos, perguntas etc. Uma peça em formato de ventarola, produzida pelo ImPrEP CAB Brasil, evoca essa perspectiva, ao anunciar a tecnologia medicamentosa, combinando imagens de comprimidos com as cores do arco-íris e as redes sociais e demais fontes eletrônicas de informação. 

**Figura 1:**
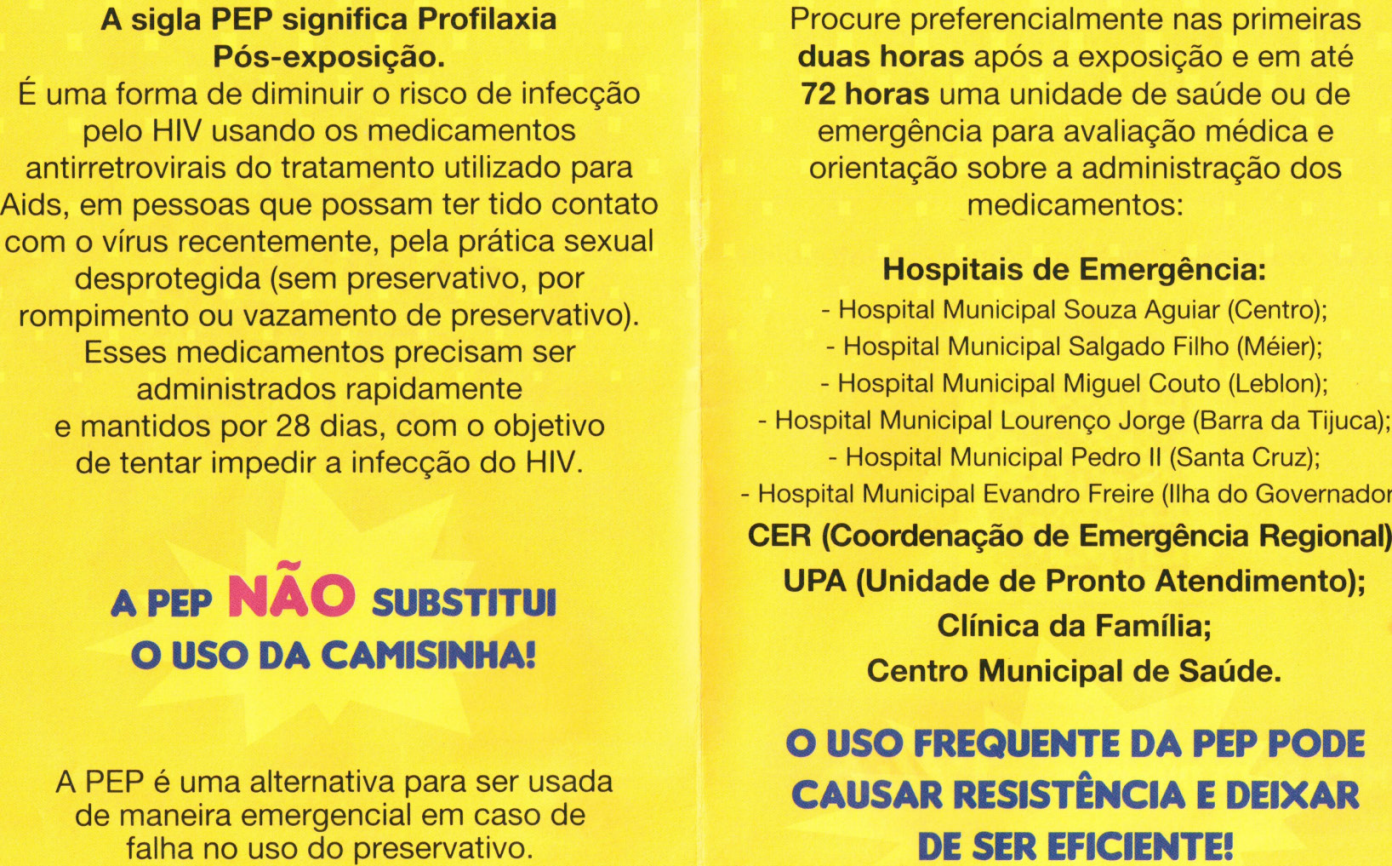
Excerto do fôlder *A Camisinha Estourou?*, da Prefeitura Municipal do Rio de Janeiro, Brasil.

Os títulos de alguns panfletos dizem respeito à convocação para participar em ensaios clínicos (*PrEP de Longa Duração*; *PreParadas*) ou focalizam nas informações específicas de cada profilaxia. Os títulos do fôlder *PEP Sexual - O Que É e Quando é Indicada* e do panfleto *Você Conhece a PrEP?* ilustram tal abordagem comunicacional. Observamos uma variação singular por meio do fôlder, produzido pela Prefeitura do Rio de Janeiro, intitulado *A Camisinha Estourou?*, que lembra aos leitores as práticas sexuais e as possibilidades de uso do insumo (Figura 1). 

**Figura 2:**
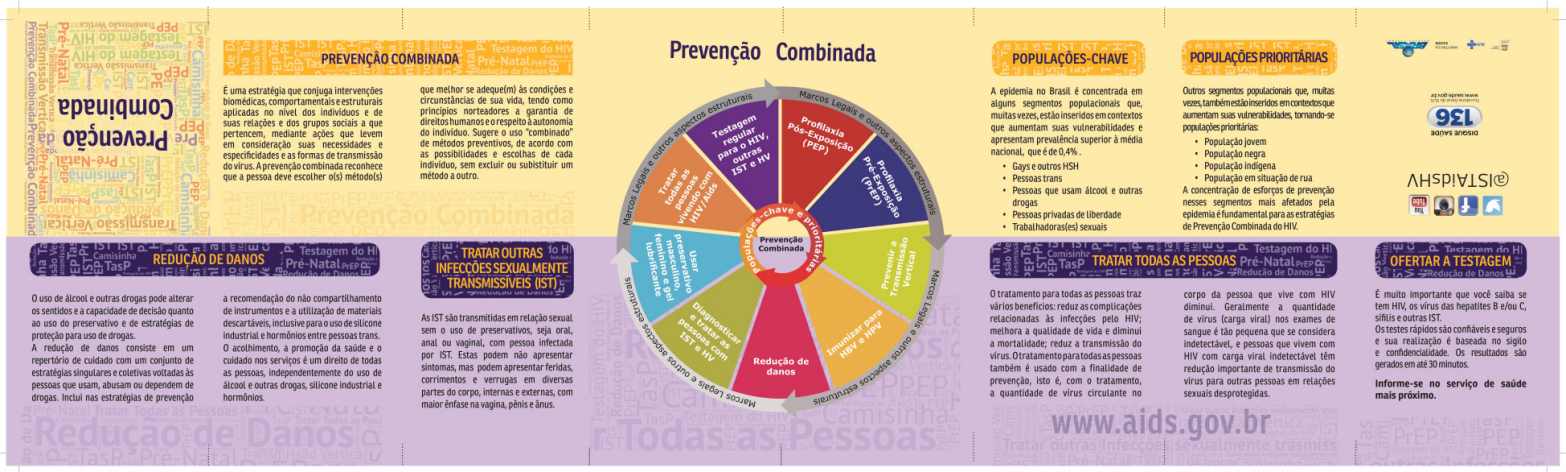
Frente do *Folder Essencial sobre Prevenção Combinada*, do Ministério da Saúde, Brasil, 2017.

A PC emerge nas peças de comunicação através da enunciação das suas três dimensões constitutivas, mas focaliza nos usos dos insumos de prevenção. Dos materiais governamentais, apenas um vocaliza a PC, trata-se do *Folder Essencial sobre Prevenção Combinada* (Figura 2).

**Figura 3:**
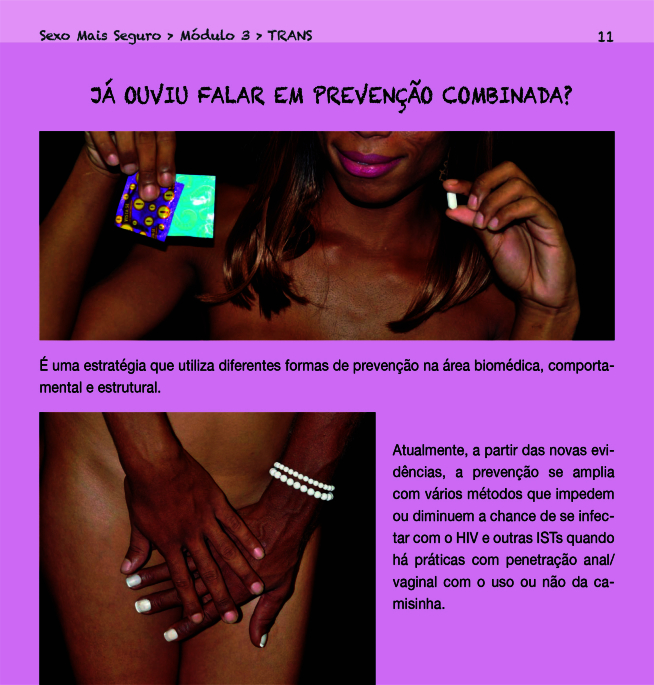
Página 11 do guia *Sexo Mais Seguro. Um Guia para Mulheres Trans e Travestis sobre Sexo, Prazer e Saúde no Século 21 para Prevenção do HIV*, da Associação Brasileira Interdisciplinar de Aids ^28^.

A maioria dos materiais produzidos pelas ONGs também tende a abordar as profilaxias em questão sem evocar a PC. Alguns contextualizam as estratégias de prevenção para os corpos, práticas e identidades sexuais dissidentes. Particularmente, o guia produzido pela Associação Brasileira Interdisciplinar de Aids (ABIA) ^28^, *Sexo Mais Seguro. Um Guia para Mulheres Trans e Travestis sobre Sexo, Prazer e Saúde no Século 21 para prevenção do HIV* além de fazer um apelo ao prazer, mobiliza a concepção original da PC (Figura 3).

**Figura 4:**
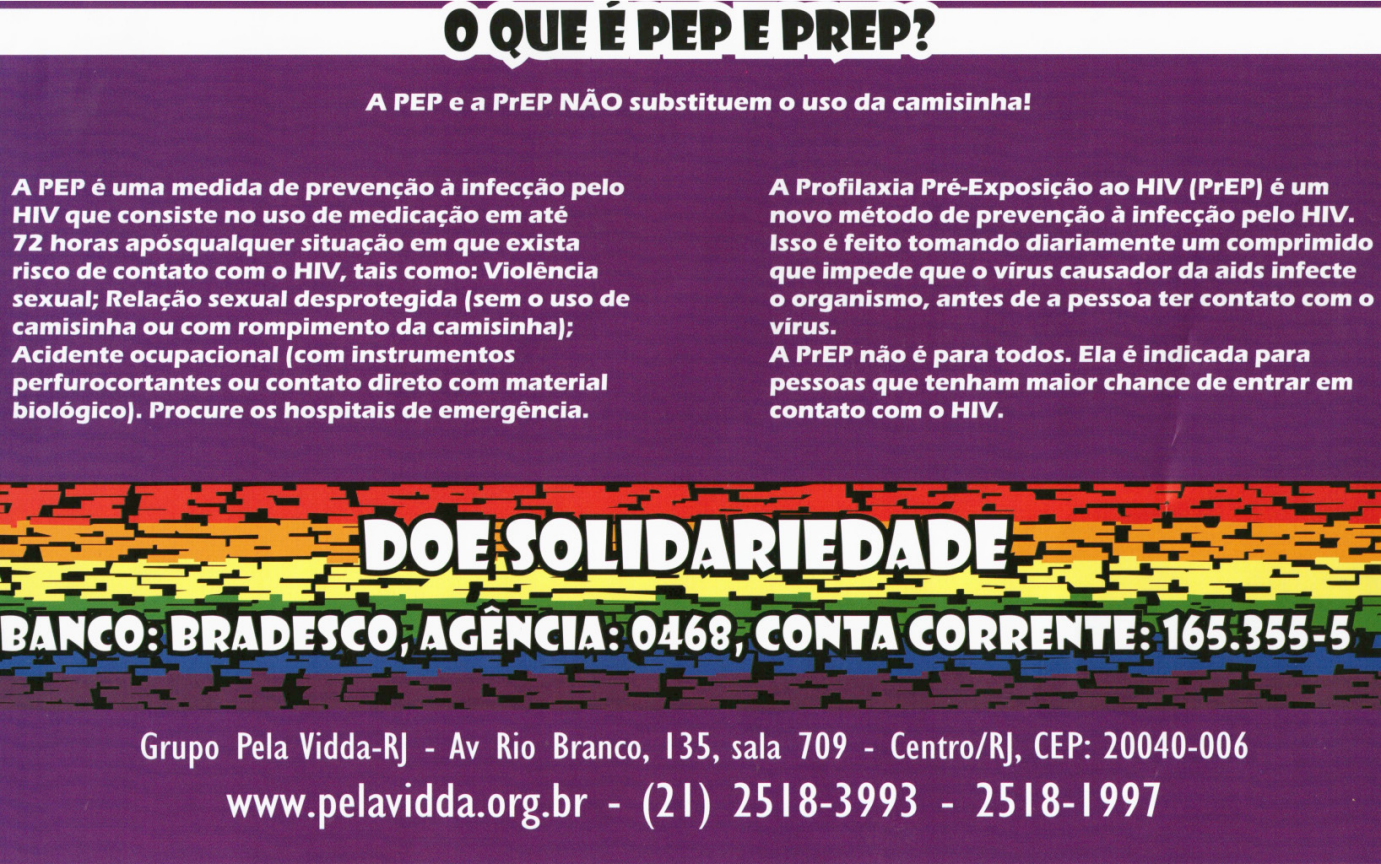
Excerto do cartaz *HIV-AIDS Quando Fazer o Teste?*, do Grupo Pela Vidda do Rio de Janeiro, Brasil*.*

Acionando outro formato e linguagem, o grupo Grupo Pela Vidda do Rio de Janeiro aborda diversos aspectos da prevenção da aids por meio do cartaz *HIV-AIDS Quando Fazer o Teste?* (Figura 4)*.* O material explica sucintamente a lógica das profilaxias e destaca um quadro comparativo sobre como se transmite e não se transmite o HIV. Esse último recurso, muito presente nos materiais comunicacionais das décadas de 1990 e 2000, é reescrito pela sociedade civil, apontando aspectos sociais que contribuem para a infecção, como o preconceito e a falta de informação.

**Figura 5:**
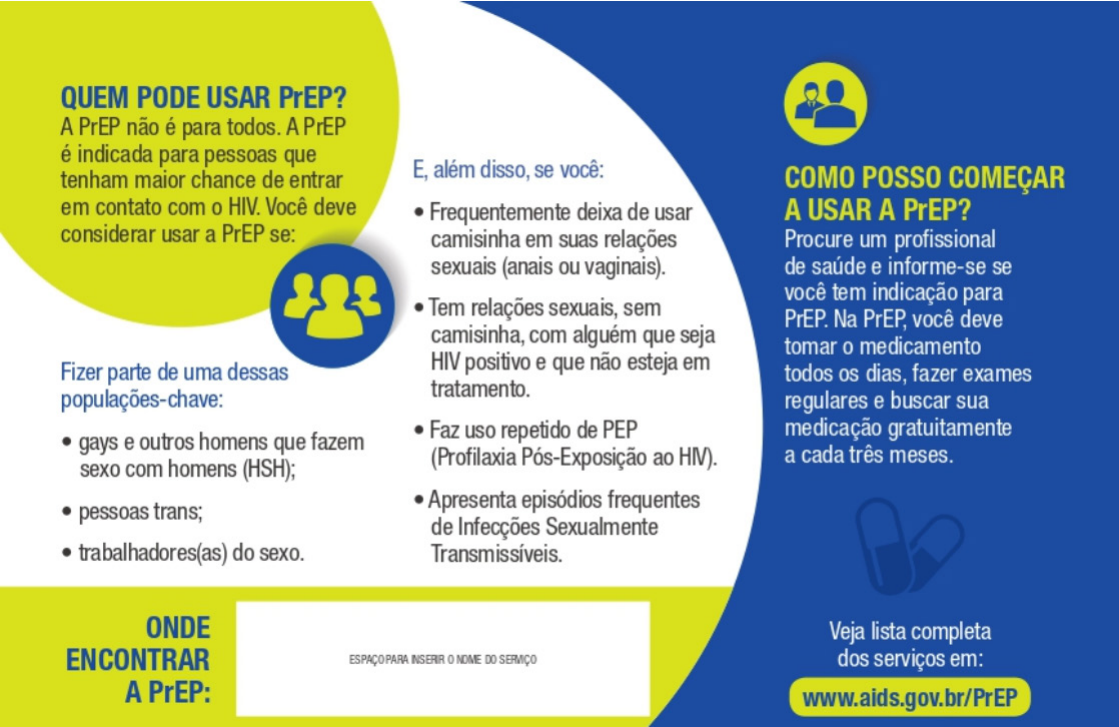
Verso do fôlder *O que é Prep?*, do Ministério da Saúde, Brasil, 2017.

Boa parte das peças analisadas incorporavam os contatos e endereços dos locais de assistência para as profilaxias, como o panfleto intitulado *O Que É Prep?*, produzido pela ONG Grupo Pela Vidda, em Niterói (Figura 5). O material se caracteriza pelo uso de frases curtas respondendo à pergunta, pontuando os objetivos, meios e regimes da profilaxia, acompanhadas das informações da ONG e do hospital parceiro na região. A diferença do fôlder produzido pelo Ministério da Saúde, também intitulado *O Que É Prep?* circunscreve a diretriz das populações chave nos seguintes termos: *A Prep Não É para Todos*; o panfleto da ONG se preocupa menos em enunciar os sujeitos alvo da estratégia e mais em ressaltar o “acolhimento” como parte da abordagem da equipe da unidade de referência.

As mensagens predominantes nas peças de comunicação, relativas às formas de enunciação dos locais de encaminhamento das profilaxias, apontam em duas direções. A primeira refere-se à constatação das alianças entre atores e instituições, seja pelo caráter novidadeiro das tecnologias ou pelos desafios da comunicação com os supostos grupos alvo das estratégias.

O segundo aborda uma lacuna entre o denominado tempo da prevenção e o tempo da prescrição, mais recorrente nos materiais governamentais. O apelo ao tempo da prevenção só é registrado nas peças quando apontados os canais de informação a serem acessados pelo público, via internet ou telefone. Práticas de diálogo em torno da PC e as experiências e necessidades dos sujeitos, independentemente dos critérios de inclusão ou exclusão das estratégias de prevenção, não são enunciados. Espaços como CTAs, unidades da atenção básica, sociedade civil ou equipamentos públicos de cidadania, presentes na exitosa resposta brasileira à aids, não são nomeados nas peças. Apenas os locais de referência, que funcionam mais sob a lógica da prescrição das profilaxias, emergem nas peças de comunicação como os únicos equipamentos que fazem prevenção. Logo, faz-se crer que só há prevenção onde há dispensação de medicamentos, num movimento de cristalização da concepção da prevenção sob a lógica dos protocolos clínicos.

## Considerações finais

O estudo objetivou compreender as concepções e práticas em torno das estratégias de comunicação da PEP e PrEP sob o ponto de vista de gestores e profissionais, envolvidas nos seus processos de implementação em municípios do Estado do Rio de Janeiro. As lacunas na dimensão da comunicação das profilaxias na esfera federal parece se reproduzir no âmbito local, dadas as imprecisões e/ou poucas referências dos interlocutores sobre o tema. Por outro lado, a produção de peças sob a rubrica das ONGs e o compartilhamento da experiência dos sujeitos com as profilaxias tem significativa importância na visibilização das estratégias, mesmo diante da atual fragilização do movimento social de aids. Ou seja, a ausência de referência às organizações sociais como agentes de divulgação das profilaxias contrasta com sua expressividade e reconhecimento por parte dos atores locais na presente análise.

Vários dos materiais produzidos pelo nível federal e instituições públicas de pesquisa privilegiam suas redes sociais ou sites nas mensagens, estimulando a procura ou ampliação das informações por parte dos interessados, como aprofundado em outro momento ^25^. São inegáveis as potencialidades das campanhas disponibilizadas via internet, em termos da diversificação de linguagens, ferramentas e poder de alcance. Entretanto, o crescente investimento em uma “*gramática biomedicalizada da prevenção ao HIV*” ^(^ ^25^ (p. 6) suscita o desafio de identificar símbolos, linguagens e canais de comunicação para reduzir a distância entre o público destinatário e as estratégias preventivas. Pesquisas assinalam as desigualdades na competência informacional e no acesso à internet entre alguns segmentos sociais, com variações importantes segundo a região e a classe no Brasil. Os achados de Avelino-Silva et al. ^29^ sobre conhecimento e acesso à prevenção biomédica apontam uma tendência ao maior domínio de informações entre homens gays e a soropositividade; ademais, assinalam que a abordagem preventiva de pessoas trans não passa por ferramentas digitais, devido ao baixo uso. 

Depreende-se que a pulverização das estratégias de comunicação constitui um traço característico das transformações no enfoque da prevenção da aids, em sintonia com as recomendações de agências internacionais, como o Programa Conjunto das Nações Unidas sobre HIV/AIDS (UNAIDS) ^12^ ^,^ ^13^. Ao que parece, as denominadas populações chave são idealizadas como unidades sociais separáveis e estanques nas suas práticas, lógicas de prevenção e subjetivação dos riscos. Predomina, assim, a visão de que a prevenção se traduz no acesso das populações chave às profilaxias.

Ao longo da resposta oficial à aids, atestamos as transformações de uma narrativa inicial, baseada no medo e nos simbolismos de uma epidemia, para um modelo centrado nos direitos humanos, que buscava incorporar os aspectos subjetivos, políticos e institucionais da comunicação, com significativos investimentos em campanhas massivas. Atualmente, notamos um deslizamento das estratégias de comunicação em direção a uma gramática informada pela disponibilidade de biotecnologias e sua enunciação dispersa, relativamente discreta, e protagonizada por múltiplas vozes, especialmente no plano digital ^30^.

No universo pesquisado, na ausência de uma narrativa pública de largo alcance, para além do arsenal biomédico, a difusão da PC é descrita como um processo árduo que resulta na pulverização das mensagens e atores da prevenção. Essa lacuna produz efeitos programáticos e simbólicos preocupantes, como o encobertamento do preconceito de alguns profissionais de saúde, dificultando a indicação da PrEP ^31^ e da PEP ^32^ somado à invisibilidade da rede de agentes e instituições, em especial, da sociedade civil, que dinamizam a circulação de informações preventivas. Essa fragilização da comunicação parece comprometer o direito à informação e à prevenção.

Os desafios da expansão das profilaxias preventivas no Brasil aumentam frente às disparidades no acesso, inclusive após o período da pandemia da COVID-19. Como revelado pelo Relatório de Monitoramento de PrEP e PEP ^33^, o predomínio do uso da PrEP entre homens gays e bissexuais com alta/média renda e escolaridade contrasta com a reduzida demanda de populações igualmente vulneráveis ao HIV, como mulheres trans, trabalhadoras sexuais, adolescentes, gays/HSH de baixo poder aquisitivo e de cor parda e preta. No caso da PEP, igualmente predomina o acesso entre homens gays e outros HSH cis (62%), com discreto incremento entre homens trans (2%) e mulheres trans e/ou travestis (4%) e declínio entre mulheres cis (33%).

Reflexões em torno dos desafios da implementação da PrEP, adicionalmente, enfatizam a necessidade de investimento em intervenções comunitárias, as quais passam pela contribuição dos educadores de par na construção de vínculos entre serviços e usuários e contemplam as redes sociais como fonte de acesso à informação e de referência ^34^. 

A diretriz do Governo Federal atual visa ampliar a dispensação da PrEP em 300% no país até 2027. Diante dos desafios desse investimento, ainda não fica claro qual será o valor atribuído aos aspectos comunicacionais e ao escalonamento da profilaxia e qual é o papel da comunicação na PC. Por fim, cumpre indagar, em análises futuras, se há uma linha de continuidade do fim da excepcionalidade da epidemia ^27^ através da reconfiguração das concepções e ações de comunicação e informação nos discursos oficiais.
